# PI3K/mTOR inhibition attenuates cigarette smoke-induced senescence and SASP in oral fibroblasts: implications for tumor microenvironment remodeling

**DOI:** 10.3389/fcell.2026.1745944

**Published:** 2026-04-29

**Authors:** Nicla Flacco, Cristina Estornut, Inés Roger, Pilar Ribera, Germán Sánchez-Herrera, Silvia Trujillo-Barbera, Martín Pérez-Leal

**Affiliations:** 1 Faculty of Health Science, Universidad Europea de Valencia, Valencia, Spain; 2 Department of Pharmacology, Faculty of Medicine, University of Valencia, Valencia, Spain; 3 Biomedical Research Networking Centre on Respiratory Diseases (CIBERES), Health Institute Carlos III, Madrid, Spain; 4 Fundación Investigación Hospital General Universitario de Valencia, Valencia, Spain; 5 Centro Avanzado de Microbiología Aplicada, Universitat Politècnica de València, Valencia, Spain

**Keywords:** cigarette smoke, oral fibroblasts, PI3K/mTor inhibition, SASP, senescence, senomorphic, tobacco, tumor microenvironment

## Abstract

**Background:**

Cigarette smoke exposure is a major risk factor for oral cancer, partly due to its ability to induce early molecular alterations in the oral mucosa. Cellular senescence and the senescence-associated secretory phenotype (SASP) contribute to chronic inflammation and microenvironmental remodeling, favoring carcinogenesis. The PI3K/AKT/mTOR pathway has been implicated in sustaining SASP and senescence, suggesting that its inhibition may represent a promising preventive strategy.

**Methods:**

Primary human oral fibroblasts (hOF) and a 3D oral mucosa model were exposed to cigarette smoke extract (CSE) 2% for 72 h, with or without pre-treatment with the dual pan–class I PI3K/mTOR inhibitor PKI402 (10^−7^-10^−9^ M). Senescence markers (p21, p16, lamin B1) were quantified by RT-qPCR; SASP cytokines (IL-6, IL-8) by ELISA; DNA damage by γH2AX immunofluorescence; and senescence-associated β-galactosidase (SA-β-gal) activity by flow cytometry.

**Results:**

CSE exposure induced a senescent phenotype characterized by increased p21 and p16, decreased lamin B1, elevated IL-6 and IL-8 secretion, accumulation of γH2AX foci, and enhanced SA-β-gal activity. PKI402 significantly attenuated these changes in a dose-dependent manner, with the highest concentrations showing statistically significant reductions across all endpoints.

**Conclusion:**

Pharmacological inhibition of PI3K/mTOR mitigates cigarette smoke-induced senescence and SASP in oral fibroblasts, reducing DNA damage and inflammatory signaling. These findings highlight PI3K/mTOR pathway as a potential target for senomorphic interventions aimed at preventing pro-tumorigenic microenvironment remodeling in oral carcinogenesis.

## 1 Introduction

Oral squamous cell carcinoma (OSCC) accounts for approximately 0.38 million new cases and around 0.17 million deaths worldwide each year (Bray et al., 2024). Tobacco smoking is the principal, modifiable risk factor for OSCC and is robustly associated with increased incidence and mortality (Jiang et al., 2019; Eloranta et al., 2024; Yang et al., 2023). Alcohol consumption and human papillomavirus (HPV) have also been linked to oral and head and neck cancers (Eloranta et al., 2024; Yang et al., 2023; Fonsêca et al., 2023). Clinically, a fraction of oral cancers arises from oral potentially malignant disorders (OPMD) such as leukoplakia and erythroplakia, with heterogeneous malignant transformation risk across lesions and sites (Warnakulasuriya et al., 2021; Pimenta-Barros et al., 2025). Therapeutically, many patients present with locally advanced disease requiring multimodal surgery and (chemo)radiotherapy, and outcomes in the recurrent/metastatic setting remain limited despite immune checkpoint inhibitors—underscoring the need for preventive, microenvironment‐modulating strategies (Imbesi Bellantoni et al., 2023; Bera et al., 2023).

Building on this disease context, cigarette smoke exposure drives early molecular alterations in the oral mucosa (Foki et al., 2020). Among these alterations, stress- and damage-induced cellular senescence has emerged as a central program, characterized by a stable cell-cycle arrest and acquisition of a pro-inflammatory secretome—the senescence-associated secretory phenotype (SASP)—that remodels the local stroma, sustains chronic inflammation, and can facilitate progression toward premalignant lesions and carcinogenesis (McHugh et al., 2025; Dong et al., 2024; Colucci et al., 2025). Senescence-related and SASP-like alterations have been described in the context of OPMD, suggesting that senescence may contribute to early stromal–epithelial remodeling in premalignant oral mucosa (Niklander et al., 2023).

Several studies have demonstrated that cigarette smoke exposure induces DNA damage, oxidative stress, and activation of signaling pathways that sustain senescence and SASP, including the PI3K/AKT/mTOR axis (Pérez-Leal et al., 2025; Nyunoya et al., 2006; Fan et al., 2025; He et al., 2024; Komaki and Ibuki, 2025). This pathway not only regulates cell survival but also plays a central role in the translation of pro-inflammatory cytokines, amplifying the impact of SASP on the tumor microenvironment (Laberge et al., 2015; Bent et al., 2016). Sustained activation of PI3K/AKT/mTOR has also been shown to perpetuate pathological senescence, promoting the secretion of factors that enhance angiogenesis, immune evasion, and malignant transformation (Belli et al., 2023; Jiang et al., 2020; Kumari and Jat, 2021). Increased expression of p21 and p16, decreased lamin B1, elevated senescence-associated β-galactosidase (SA-β-gal) activity, and accumulation of γH2AX foci indicative of DNA damage are markers associated with senescence; in parallel, cytokines such as IL-6 and IL-8 are commonly associated with SASP (Xiao et al., 2024). In previous studies by our group, we confirmed that cigarette smoke induces senescence and SASP markers in human oral mucosa, reinforcing the relevance of this pathway in early carcinogenic processes (Pérez-Leal et al., 2025).

Therefore, inhibition of the PI3K/mTOR axis has been proposed as a potential strategy to counteract cigarette smoke-induced senescence and limit its pro-carcinogenic impact in chronically exposed tissues such as oral mucosa (Peng et al., 2022). In this study, we evaluated the effect of the dual pan–class I PI3K/mTOR inhibitor PKI402 on human oral fibroblasts exposed to cigarette smoke extract (CSE). PKI402 is a research-grade, dual pan-class I PI3K/mTOR inhibitor widely used in preclinical models (Mallon et al., 2010). Accordingly, this work aims to evaluate axis-level, senomorphic effects on CSE-induced senescence and SASP through PI3K/mTOR inhibition in oral fibroblasts, within a predefined mechanistic *in-vitro* scope. The novelty of this work lies in a microenvironment-centric, axis-level (PI3K/mTOR) senomorphic approach in primary human oral fibroblasts, using an orthogonal, rigor-oriented senescence/SASP panel under cigarette smoke exposure.

## 2 Materials and methods

### 2.1 Cell culture and treatments

Primary human oral fibroblasts (hOF) were obtained from ScienCell Research Laboratories (Carlsbad, CA, United States), certified as mycoplasma-free. Cells were cultured in Fibroblast Medium (ScienCell) supplemented with 2% fetal bovine serum (FBS), 1% fibroblast growth supplement, and 1% penicillin/streptomycin solution. Cultures were maintained in a humidified incubator at 37 °C with 5% CO_2_. Cells between passages 2 and 4 were used to minimize phenotypic drift and spontaneous senescence associated with higher passages (Pérez-Leal et al., 2025). Cultures were selected based on typical fibroblast morphology, characterized by spindle-shaped, elongated cells with homogeneous distribution and absence of vacuolization or detachment, as assessed by phase-contrast microscopy, together with maintained cell viability.

For the three-dimensional (3D) model, reconstructed human oral epithelium SkinEthic™ HOE (EPISKIN, Lyon, France) was used, which mimics stratified oral mucosa. The model was cultured for 5 days in SkinEthic Maintenance Medium at 37 °C, 5% CO_2_, and 95% humidity to allow epithelial layer development.

Cigarette smoke extract (CSE) was prepared using 1R6F reference cigarettes (Kentucky Reference Cigarettes). Smoke from three cigarettes was generated using a rodent respirator (Rodent Respirator 680, Harvard Apparatus) and bubbled through 25 mL of pre-warmed RPMI medium (37 °C). The extract was filtered through 0.22 μm syringe filters (Merck Millipore) to remove particulates and considered 100% CSE stock solution. This was diluted in Fibroblast Medium to a final concentration of 2%. Cells were exposed to CSE for 72 h to simulate sustained/chronic exposure conditions, as commonly used in previous studies evaluating cigarette smoke-induced senescence and SASP responses *in vitro* (Pérez-Leal et al., 2025).

The dual pan–class I PI3K/mTOR inhibitor PKI402 was added 1 h prior to CSE exposure at concentrations of 10^−7^ M, 10^−8^ and 10^−9^ M, diluted in Fibroblast Medium. In the SkinEthic™ HOE 3D, PKI402 was applied at 10^−7^ M in Maintenance Medium, preselected from the 2D assays as the highest concentration yielding significant attenuation across senescence and SASP endpoints. The use of a single concentration in the 3D model was based on its effectiveness in 2D conditions and on practical constraints related to the limited number of inserts. No medium change was performed during the 72 h exposure.

### 2.2 qPCR

Total RNA was extracted from cell cultures and the 3D model using TRIzol™ reagent (Invitrogen), following the manufacturer’s standard protocol. Samples were homogenized in TRIzol, followed by phase separation with chloroform. The aqueous phase was carefully recovered, and RNA was precipitated with isopropanol. The RNA pellet was washed with 75% ethanol, air-dried, and resuspended in nuclease-free water.

RNA concentration and purity were assessed using a NanoDrop spectrophotometer (Thermo Fisher Scientific), ensuring an appropriate A260/A280 ratio for reverse transcription. cDNA was synthesized from 500 ng of RNA using the High-Capacity cDNA Reverse Transcription Kit (Applied Biosystems) and stored at −20 °C until use.

Gene expression was quantified by real-time PCR (RT-qPCR) using TaqMan Gene Expression Assays (Applied Biosystems) for CDKN1A (p21; assay ID: Hs00355782_m1), CDKN2A (p16; assay ID: Hs00923894_m1), and LMNB1 (lamin B1; assay ID: Hs01059205_m1). GAPDH (assay ID: Hs02786624_g1) was used as the reference gene for normalization. Reactions were performed in 384-well plates using the QuantStudio™ 5 system (Applied Biosystems), with a final volume of 10 μL per well. Each sample was analyzed in technical duplicates, and no-template controls were included. Relative expression levels were calculated using the 2^−ΔΔCt^ method, comparing values to the untreated control group. Results were expressed as fold change relative to control.

### 2.3 ELISA (enzyme-linked immunosorbent assay)

Levels of IL-6 and IL-8 in culture supernatants were quantified using commercial ELISA kits (Invitrogen, Thermo Fisher Scientific, Waltham, MA, United States), following the manufacturer’s instructions. Supernatants were collected after 72 h of exposure to 2% CSE and stored at −80 °C until analysis.

Assays were performed in duplicate for each sample. Each kit included 96-well plates pre-coated with capture antibodies specific for IL-6 and IL-8, and a horseradish peroxidase (HRP)-conjugated detection antibody. After sample addition, plates were incubated at room temperature for 2 h, followed by washing and incubation with the detection antibody for 1 h. HRP substrate was then added and incubated for 30 min, and the reaction was stopped using the provided stop solution.

Absorbance was measured at 450 nm using a SpectraMax iD3 plate reader (Molecular Devices, San Jose, CA, United States). Cytokine concentrations were calculated from a standard curve generated with known concentrations of recombinant cytokines provided in the kits. To account for condition-dependent differences in viable cell content, cytokine values were expressed in picograms per milliliter (pg/mL) and were normalized to percentage of cell viability measured by the MTT assay (A570-A630).

### 2.4 Fluorescence microscopy for DNA damage

DNA damage was assessed by immunofluorescence detection of γH2AX, a marker of double-strand breaks. After treatments, fibroblasts were fixed with 4% paraformaldehyde for 15 min at room temperature and permeabilized with 0.1% Triton X-100 in PBS for 10 min. Cells were blocked with 1% BSA in PBS for 30 min and incubated overnight at 4 °C with anti-γH2AX primary antibody (recombinant monoclonal, clone BLR053F, Thermo Fisher Scientific, ref. A700-053) diluted 1:500 in PBS with 1% BSA.

Cells were then incubated with Alexa Fluor™ 594-conjugated secondary antibody (Thermo Fisher Scientific) for 1 h at room temperature in the dark. Nuclei were counterstained with DAPI (0.5 μg/mL) for 5 min. Samples were mounted with antifade medium and visualized using a Leica DMi8 fluorescence microscope. Representative images were captured under identical acquisition settings. Quantification of γH2AX signal was performed using CellProfiler software by measuring the mean fluorescence intensity within nuclear regions of interest (ROIs) defined by DAPI staining. The same analysis pipeline, including segmentation parameters and intensity thresholds, was applied uniformly across all images to ensure comparability between conditions.

### 2.5 Flow cytometry

To assess SA-β-gal activity, hOF were cultured and exposed to 2% CSE for 72 h. Cells were then incubated with a fluorescent substrate specific for SA-β-gal, following the manufacturer’s instructions.

Analysis was performed using a BD FACSVerse™ flow cytometer (BD Biosciences). Data were acquired and analyzed using BD FACSDiva software. Events were initially gated on forward scatter area (FSC-A) versus side scatter area (SSC-A) to exclude debris. Singlet populations were identified using FSC-A versus FSC-H. SA-β-gal activity was detected in the FITC-A channel, and the population of interest (P3) was defined based on FSC-A versus FITC-A, selecting viable cells with detectable fluorescence.

The prespecified primary endpoint was the mean fluorescence intensity (MFI) in FITC-A within the P3 population. For an objective definition of SA-β-gal–positive events, a control-based threshold was set to the 95th percentile of the FITC-A distribution in the untreated control acquired in the same run and applied uniformly across conditions within that experiment. Flow cytometry plots were exported directly from BD FACSDiva without data modification.

## 3 Results

### 3.1 Gene expression of senescence markers

To assess the preventive effect of PI3K/mTOR inhibition on cigarette smoke-induced senescence and SASP, RT-qPCR analysis was performed in human oral fibroblasts (hOF) and the 3D oral mucosa model. Exposure to 2% cigarette smoke extract (CSE) for 72 h significantly increased the expression of p21 and p16, accompanied by a decrease in lamin B1 levels compared to the control group. Treatment with PKI402 attenuated these changes in a dose-dependent manner. Results were similar and comparable in the monolayer culture and in the 3D culture, for the dose used of 10^−7^ M. This approach is consistent with the increased structural complexity and reduced drug penetration typically observed in 3D models compared to monolayer cultures.

**FIGURE 1:**
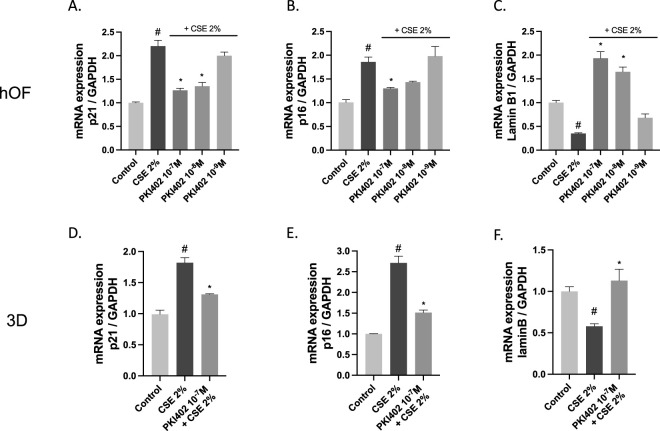
RT-qPCR analysis of senescence markers in human oral fibroblasts (hOF) and 3D oral epithelium cultures treated with 2% cigarette smoke extract (CSE) for 72 h, with or without PKI402 at 10^−7^, 10^−8^ and 10^−9^ M in hOF and 10^−7^ M in the 3D model. Relative gene expression levels of p21 **(A,D)**, p16 **(B,E)**, and lamin B1 **(C,F)** are shown, normalized to GAPDH and expressed as fold change relative to the untreated control. Results are presented as mean ± standard error of the mean from three independent experiments (n = 3). Statistical significance was determined using one-way ANOVA followed by Tukey’s *post hoc* test. #p < 0.05 vs. control; *p < 0.05 vs. CSE-treated group.

The panels are as follows: in hOF, p21 is shown in Figure 1A, p16 in Figure 1B, and lamin B1 in Figure 1C; in the 3D model, p21 in Figure 1D, p16 in Figure 1E, and lamin B1 in Figure 1F. Across panels A–F, CSE increases p21 and p16 while decreasing lamin B1, and PKI402 mitigates these effects in a dose-dependent manner, with the largest effects at 10^−7^ and 10^−8^ M (p < 0.05).

### 3.2 SASP cytokine secretion

**FIGURE 2:**
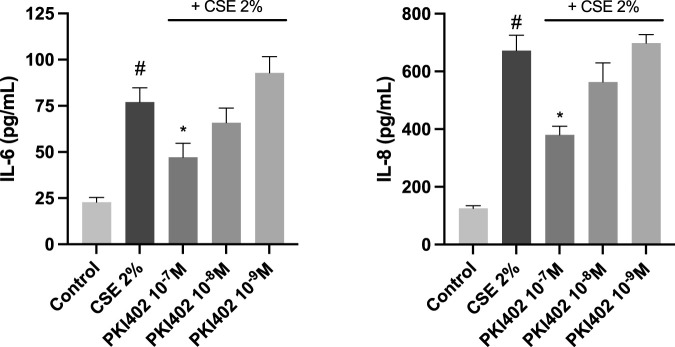
Quantification of pro-inflammatory SASP-associated cytokines (IL-6 and IL-8) in the supernatant of human oral fibroblasts treated with 2% cigarette smoke extract (CSE) for 72 h, with or without PKI402 (10^−7^-10^−9^ M). Cytokine levels were determined by ELISA, and normalized to percentage of cell viability, values are expressed as pg/mL. Results are presented as mean ± standard error of the mean from at least three independent experiments (n = 3). Statistical significance was assessed using one-way ANOVA followed by Tukey’s *post hoc* test. #p < 0.05 vs. control; *p < 0.05 vs. CSE-treated group.

Quantification of IL-6 and IL-8 by ELISA revealed a significant increase in both cytokines following CSE exposure in hOF. Treatment with PKI402 reduced IL-6 and IL-8 secretion at all tested concentrations, with statistically significant reductions observed at 10^−7^ M compared to the CSE group (p < 0.05) (Figure 2), consistent with attenuation of the SASP phenotype observed in gene-expression analyses. To control for differences in viable cell content, ELISA values were normalized to cell viability measured by MTT. The same pattern of changes was observed after normalization.

### 3.3 DNA damage

**FIGURE 3:**
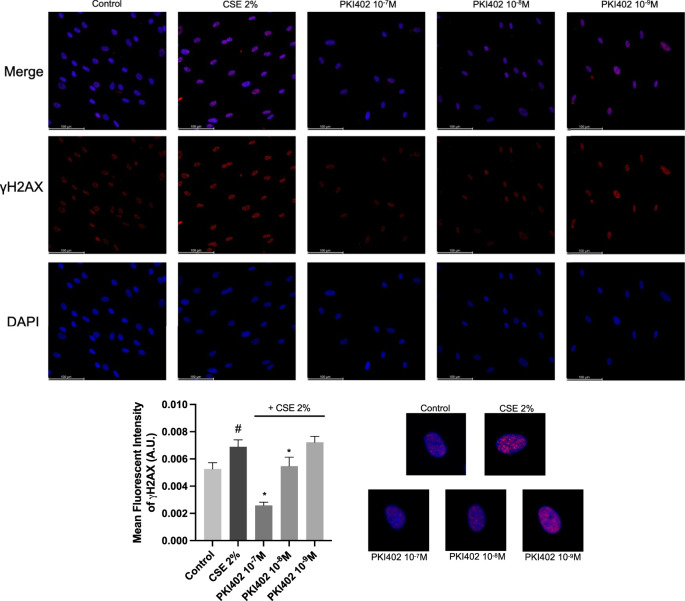
Detection of DNA damage by γH2AX immunofluorescence in human oral fibroblasts treated with 2% cigarette smoke extract (CSE) for 72 h, with or without PKI402 (10^−7^-10^−9^ M). Representative images show γH2AX staining (red), nuclei counterstained with DAPI (blue), and merged images. The lower panel displays the quantification of the mean γH2AX fluorescence intensity. To the right of the lower panel, an enlarged merged nuclear inset is provided for each condition to facilitate visual assessment of γH2AX puncta. Results are presented as mean ± standard error of the mean from three independent images. Statistical significance was assessed using one-way ANOVA followed by Tukey’s *post hoc* test. #p < 0.05 vs. control; *p < 0.05 vs. CSE-treated group. A.U.: Arbitrary Units.

Immunofluorescence analysis of γH2AX revealed a marked increase in nuclear γH2AX fluorescence in fibroblasts exposed to 2% CSE (Figure 3). Treatment with PKI402 significantly reduced the intensity of γH2AX foci at 10^−7^ M and 10^−8^ M compared with the CSE group (p < 0.05) (Figure 3). γH2AX is employed as a sensitive marker of double-strand DNA breaks, reflecting activation of the DNA damage response. Quantification based on mean fluorescence intensity was selected to capture overall changes in γH2AX signal per nucleus, reflecting the extent of DNA damage. This approach allows detection of graded changes in DNA damage levels that may not be captured by simple counting of positive nuclei. CSE exposure promoted a significant increase in the mean γH2AX fluorescence intensity, indicating accumulation of DNA damage. Conversely, treatment with PKI402 effectively prevented cigarette-induced DNA damage, reducing γH2AX signal and indicating a protective effect on genomic integrity at these doses. To aid visual interpretation, the right-hand side of Figure 3 includes an enlarged merged (γH2AX/DAPI) nuclear inset for each condition, highlighting the increase in γH2AX puncta after CSE and their reduction following PKI402 treatment.

### 3.4 Senescence activity

**FIGURE 4:**
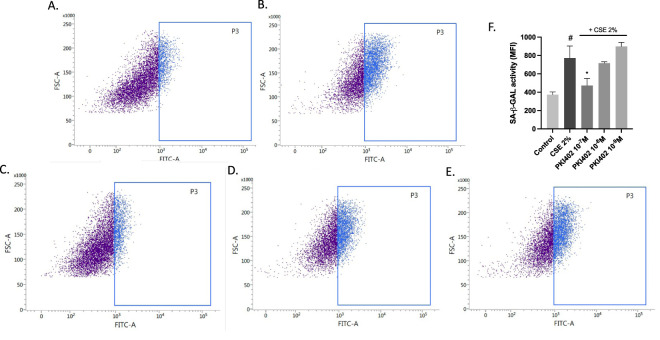
Assessment of senescence activity by flow cytometry in human oral fibroblasts treated with 2% cigarette smoke extract (CSE) for 72 h, with or without PKI402 (10^−7^-10^−9^ M). Representative histograms of SA-β-gal-associated fluorescence are shown (**(A)** Control; **(B)** CSE 2%; **(C)** PKI402 10^−7^ M; **(D)** PKI402 10^−8^ M; **(E)** PKI402 10^−9^ M), along with quantification of mean fluorescence intensity in the viable cell population (P3) **(F)**. Data are presented as mean ± standard error of the mean from at least three replicates (n = 3). Statistical significance was assessed using one-way ANOVA followed by Tukey’s *post hoc* test. #p < 0.05 vs. control; *p < 0.05 vs. CSE-treated group. FITC-A, fluorescein isothiocyanate—area; FSC-A, forward scatter area.

Flow cytometry analysis revealed an increase in SA-β-gal-associated fluorescence in fibroblasts exposed to CSE (Figure 4). Figure 4A shows the control cells and Figure 4B shows cells treated with 2% CSE. Treatment with PKI402 reduced fluorescence intensity in the viable cell population (P3), with statistically significant reductions observed at 10^−7^ M and 10^−8^ M (p < 0.05) (Figures 4C–E), consistent with attenuation of the senescent phenotype. Figure 4F presents the quantification of the mean fluorescence intensity (MFI) in the P3 population, confirming the increase with CSE and its dose dependent changes with PKI402 treatments.

## 4 Discussion

Exposure of human oral fibroblasts and the 3D oral mucosa model to 2% cigarette smoke extract (CSE) for 72 h induced a senescent phenotype characterized by increased expression of p21 and p16, decreased lamin B1 levels, elevated secretion of IL-6 and IL-8, increased γH2AX foci, and enhanced SA-β-gal activity. Treatment with PKI402 reversed these changes in a dose-dependent manner, with statistically significant reductions at higher concentrations. These findings provide experimental evidence that PI3K/mTOR axis inhibition attenuates cigarette-induced senescence and SASP, reduces genomic damage, and suggests a potential preventive role in tumor microenvironment remodeling associated with oral carcinogenesis. In addition, within the therapeutic landscape of head and neck squamous cell carcinoma (HNSCC), including oral cavity tumors, the PI3K/AKT/mTOR pathway is among the most frequently dysregulated axes, which further supports the biological plausibility of modulating senescence/SASP through its inhibition (Laberge et al., 2015; Belli et al., 2023). Because PKI402 is a dual pan–class I PI3K/mTOR inhibitor, we interpret these effects at the axis level and do not attribute them to PI3K or mTOR individually. Beyond reproducing pathway inhibition, the novelty of this work lies in its microenvironment-centric design, together with orthogonal, rigor-oriented endpoints that reveal axis-level senomorphic attenuation of CSE-induced senescence/SASP.

These results are consistent with existing evidence describing senescence as an initially protective mechanism that acquires pro-tumorigenic properties when sustained through SASP. In the oral mucosa, cigarette smoke has been associated with DNA damage, oxidative stress, and overexpression of p21 and p16, along with IL-6 and IL-8 secretion, contributing to chronic inflammation and progression toward premalignant lesions (Dong et al., 2024; Nyunoya et al., 2006). Recent reviews have also highlighted the central role of the PI3K/AKT/mTOR pathway in SASP regulation and tumor microenvironment remodeling (Laberge et al., 2015; Bent et al., 2016).

The attenuation of SASP and genomic damage observed following PI3K/mTOR axis inhibition aligns with the role of this pathway in mTOR-mediated translation of pro-inflammatory cytokines, including IL-1α, which amplifies NF-κB signaling and sustains the senescence-associated secretome (Laberge et al., 2015; Bent et al., 2016). In related models, PI3K/AKT/mTOR activation by miR-21, via PTEN repression, has been linked to cigarette-induced endothelial senescence, while its inhibition reverses cellular dysfunction and reduces SASP expression (He et al., 2024; Yamauchi and Takahashi, 2025). Similarly, the accumulation of γH2AX foci in our study is consistent with persistent activation of the DNA damage response (DDR), a key driver of senescence and SASP (Yamauchi and Takahashi, 2025; Osipov et al., 2023; Ngoi et al., 2021). A slight reduction in nuclei number was observed in PKI402-treated conditions, particularly at the highest concentration, which may reflect a modest effect on cell proliferation or viability. Cytokine data were normalized to cell viability (MTT), supporting that SASP changes are not solely due to differences in cell number. In this context, although NRF2 was not examined experimentally here, we note as contextual background that NRF2 can modulate senescence and SASP in fibroblasts—promoting matrisome remodeling and CAF-like programs in a context-dependent manner—and has been linked to epithelial aging phenotypes in skin models. These observations motivate future experiments to test potential NRF2–PI3K/AKT/mTOR crosstalk in oral mucosa (Hiebert et al., 2018; Zhang et al., 2024; Nan et al., 2025).

From a translational perspective, PI3K inhibition has been explored in HNSCC with both isoform-selective and pan-class I agents. PI3Kα-selective alpelisib has shown signals of activity in molecularly enriched subsets—particularly in tumors harboring activating PIK3CA mutations—although overall benefit is context-dependent and can be limited by on-target metabolic toxicity [35–37]. By contrast, pan-class I inhibitors such as buparlisib have achieved, at best, modest efficacy with tolerability constraints, driving biomarker-guided selection and rational combinations [22,38]. Within this landscape, PKI402 is a research-grade dual pan-class I PI3K/mTOR inhibitor; accordingly, our findings should be interpreted as senomorphic, axis-level mechanistic *in-vitro* observations—not evidence of clinical efficacy—and will require *in vivo* validation and dose–toxicity studies [27,20]. In this context, PKI402 was selected as a dual pan–class I PI3K/mTOR inhibitor to achieve axis-level inhibition, which may be particularly relevant for senescence and SASP regulation, given the role of mTOR in the translation of pro-inflammatory mediators. This approach enables a more comprehensive blockade of the pathway compared to isoform-selective inhibitors, supporting its use in mechanistic studies.

The use of oral fibroblasts is particularly relevant given their role in stromal signaling and paracrine propagation of SASP, modulating the extracellular matrix and local inflammation. Previous studies have shown that cigarette smoke induces senescence in fibroblasts across various tissues (Pérez-Leal et al., 2025; Nyunoya et al., 2006). Moreover, we included the SkinEthic™ HOE three-dimensional reconstructed human oral epithelium, a standardized air-liquid-interface (ALI) model that histologically resembles non-keratinized oral mucosa and is widely validated for oral toxicity and smoke-exposure studies (Klausner et al., 2021). This epithelial compartment complements our fibroblast findings by allowing direct smoke exposure at the mucosal surface under ALI conditions and by capturing epithelial-specific stress and barrier-relevant responses, thereby improving translatability to *in vivo* buccal mucosa (Schlage et al., 2014). Notably, in previous work, CSE-induced changes in oral keratinocytes were comparable to those observed in fibroblasts, both in p21 and p16 expression and SASP activation (Pérez-Leal et al., 2025). Our results are in line with recent studies showing that activation of the PI3K/AKT pathway in fibroblasts, particularly via AKT3, can promote a CAF-like phenotype capable of remodeling the tumor microenvironment in HNSCC (Takahashi et al., 2021). Reversal of SASP through PI3K/mTOR inhibition may represent a complementary strategy to limit pro-tumorigenic stromal activation during early stages of oral carcinogenesis. Within this framework, PKI402 should be interpreted as a research-grade compound rather than a clinical drug; its concurrent mTOR blockade is consistent with the “senomorphic” effects observed here, given mTOR’s role in the translation of key SASP mediators and in the NF-κB amplification loop (Laberge et al., 2015; Zhang et al., 2025).

The observed reduction in SASP and genomic damage through PI3K/mTOR axis inhibition suggest a senomorphic strategy with potential to mitigate chronic inflammation and pro-tumorigenic remodeling during early oral carcinogenesis. This approach aligns with the growing interest in senescence-targeted therapies, including senolytics and SASP modulators, which could be combined with PI3K/mTOR inhibitors to prevent progression of premalignant lesions (McHugh et al., 2025; Liu et al., 2025). Moreover, interventions that block pro-inflammatory signaling and PI3K/mTOR activity have shown efficacy in reducing cigarette-induced senescence in other tissues, further supporting the translational relevance of our findings (Khan et al., 2024).

Limitations and future directions. The main limitations of this study include the exclusive use of oral fibroblasts, as well as the lack of *in vivo* models and global transcriptomic analysis to comprehensively characterize the PI3K-mTOR-NF-κB network and its interaction with DDR pathways. Going forward, it will be important to expand experiments in 3D, incorporate epithelial–stromal co-cultures and other advanced 3D models, and reinforce the readouts with protein-level validations and mRNA–protein correlations. In the epithelial compartment, adding histology and quantitative image analysis with systematic micrographs will better complement the findings. In addition, omics approaches will help explore the heterogeneity and context-specific regulation of senescence and SASP. Accordingly, node-specific attribution (PI3K-only or mTOR-only) lies beyond the predefined scope of this pilot and will be addressed in follow-up deconvolution studies. From a translational standpoint, these findings should be considered mechanistic and restricted to *in vitro* models; *in vivo* validation with assessment of safety, dose, and pharmacodynamics will be required before any clinical inference.

## 5 Conclusion

This study shows that PI3K/mTOR inhibition attenuates cigarette smoke-induced senescence and its associated SASP in human oral fibroblasts. These findings indicate that targeting PI3K/mTOR pathway may serve as a senomorphic approach to limit early pro-inflammatory remodeling of the oral microenvironment linked to carcinogenesis.

## Data Availability

The datasets generated for this study are publicly available in the Zenodo repository: https://doi.org/10.5281/zenodo.19822370.
